# The Effect of the Nonlinearity of the Response of Lipid Membranes to Voltage Perturbations on the Interpretation of Their Electrical Properties. A New Theoretical Description

**DOI:** 10.3390/membranes5040495

**Published:** 2015-09-25

**Authors:** Lars D. Mosgaard, Karis A. Zecchi, Thomas Heimburg, Rima Budvytyte

**Affiliations:** Niels Bohr Institute, University of Copenhagen, Blegdamsvej 17, Copenhagen 2100, Denmark; E-Mails: l.d.mosgaard@gmail.com (L.D.M.); zecchi@nbi.dk (K.A.Z.); theimbu@nbi.dk(T.H.)

**Keywords:** lipid membrane, electrophysiology, capacitance, conduction, inductance, impedance

## Abstract

Our understanding of the electrical properties of cell membranes is derived from experiments where the membrane is exposed to a perturbation (in the form of a time-dependent voltage or current change) and information is extracted from the measured output. The interpretation of such electrical recordings consists in finding an electronic equivalent that would show the same or similar response as the biological system. In general, however, there is no unique circuit configuration, which can explain a single electrical recording and the choice of an electric model for a biological system is based on complementary information (most commonly structural information) of the system investigated. Most of the electrophysiological data on cell membranes address the functional role of protein channels while assuming that the lipid matrix is an insulator with constant capacitance. However, close to their melting transition the lipid bilayers are no inert insulators. Their conductivity and their capacitance are nonlinear functions of both voltage, area and volume density. This has to be considered when interpreting electrical data. Here we show how electric data commonly interpreted as gating currents of proteins and inductance can be explained by the nonlinear dynamics of the lipid matrix itself.

## 1. Introduction

In electrophysiology, the properties of ion channels and thus the explanation of the propagation of nerve signals are derived from current clamp and voltage clamp techniques. By employing voltage or current clamp, the electrical properties of a cell membrane or a lipid membrane can be investigated. For instance, the voltage clamp technique enabled Huxley and Hodgkin to characterize the electrical response of the giant squid axon to perturbations in voltage. The results lead to the development of an electrical model for nerve pulse propagation [1,2]. They assumed, as it is now customary in the field, that the lipid membrane acts as a simple constant capacitor and that the observed nonlinear currents are due to protein ion-channels which are embedded in an otherwise inert membrane. However, it has been shown that the membrane rather behaves as a nonlinear capacitor [3,4,5,6]. This potentially alters the interpretation of the electrical response of the membrane. Furthermore, it has been observed that even in the absence of proteins synthetic lipid membranes in their melting regime can display quantized conduction events that are virtually indistinguishable from those of protein channels [7]. These “lipid ion channels” behave like voltage-gated, temperature-gated, and mechano-sensitive protein channels, or like receptors [8]. In this respect, the lipid membrane itself is a nonlinear conductor. These important phenomena should be taken into account in the discussion and description of the electrical properties of membranes.

**Figure 1:**
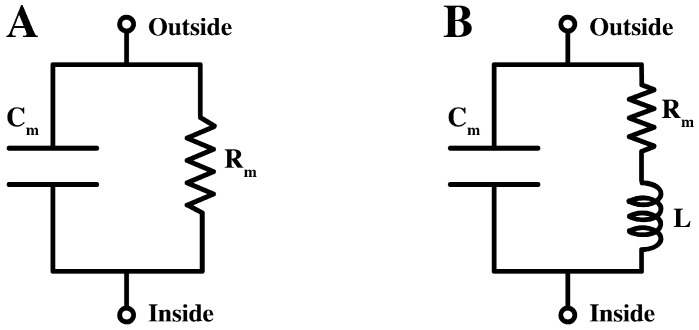
(**A**).The equivalent circuit of the lipid membrane, containing a resistor (Rm) and a capacitor (Cm) in parallel. (**B**). An approximate equivalent circuit for the membrane of the squid giant axon with additional inductance, L.

Commonly, the pure lipid membrane is considered as an insulator separating two electrically conductive compartments. The equivalent circuit of lipid bilayers takes the form of a capacitor in parallel with a resistor, see Figure 1A. In the case of biological membranes, the equivalent circuit is very similar, with the resistive branch describing the conduction of ions through specific protein channel. Back in the 1940s, however, Cole and others had presented experimental evidences for a high inductance element of 0.2 H cm${}^{2}$ in the membrane of the squid giant axon [9,10]. Quoting Cole, an inductance is generally *“a characteristic associated only with the storage of energy in a magnetic field, and it is singularly difficult to imagine a membrane structure, which would allow an electromagnetic field corresponding to more than a few microhenries”* [9]. An approximate equivalent circuit for the membrane was suggested [11] (see Figure 1B).

Lacking clear structural evidence, an inductance in the membrane was a controversial concept hard to get accustomed to by the electrophysiology community. Hodgkin and Rushton (1946) [12] thought that inductance might have been a precursor of excitation. Lorente de Nó (1947) [13] was critical and rejected it as a too linear concept. Hodgkin (1951) [14] accepted the idea and suggested that it was the result from changes of the potassium permeability [15]. Thus, the inductance was not considered as an element of such circuit. It was rather suggested that a nonlinearity in another electrical element lead to the appearance of an “inductance” [15]. Opatowski (1950) [16] asserted that inductance has an influence on the conduction velocity in the nerve fiber.

**Figure 2:**
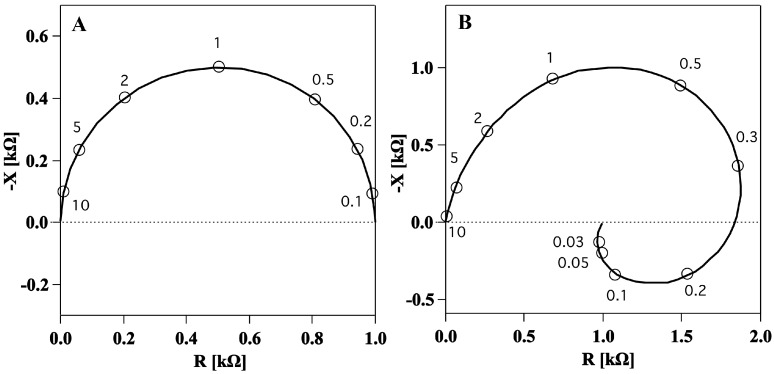
Longitudinal impedance data shown in a complex plot (Nyquist plot) showing the negative of the imaginary part (y axis) and the real part (x axis) of the impedance for different values of the frequency (open circles). (**A**). Calculated impedance spectrum of ideal capacitance resistance membrane like the one in Figure 1A. Frequencies are given in multiples of the characteristic frequency. (**B**). Measured impedance data for squid giant axon membrane in a frequency range from 10 kHz down to 30 Hz indicating an inductive element in the electrical circuit. Figures adapted from [18].

The first evidence of a membrane inductance was obtained by impedance spectroscopy, a technique which measures the electrical impedance of a system at different frequencies. The impedance of the squid giant axon was first measured by Cole and Hodgkin in 1939 [17] for a frequency range above 200 Hz. It was found to be similar to the semicircle in Figure 2A. Later, a few sets of measurements over the complete frequency range were made primarily to determine the lowest frequency over available range [11] (Figure 2B). Surprisingly, impedance spectra showed two frequency dependent elements in the membrane: one, positive, capacitive, at high frequencies and another, negative, inductive, at low frequencies (Figure 2B).

Experimentally, it was shown that neither axoplasm nor the connective tissue were responsible for the inductance. Thus, it was concluded that it was a characteristic of the membrane itself [18]. In 1942 Cole and Marmont observed that inductance effects were increased at high external potassium concentration and in the absence or at low concentration of external calcium [19]. The presence of the relatively high inductance in a small structure such as an axon membrane has been explained by different theories: (A) The electromagnetic theory; The inductance could have an electromagnetic origin. However, the membrane is too thin to account for the storage of such a high electromagnetic field [9,18]; (B) The electrodiffusion theory; The axon membrane has a nonlinear conductance, which was attributed to a flow of potassium ions. The inductive response was attributed to the time interval the ions need to penetrate the membrane [18,20]. Extensive studies of this effect include those by Chandler *et al*. (1962) and Mauro *et al*. (1970) [21,22]. Offner (1969) [23] related the membrane reactance to the dynamics of internal ions; (C) The electrostrictive or piezoelectric theory; The first consideration that membranes might have piezoelectric properties dates back 74 years to a discussion of the possible origin of the inductance in the squid giant axon [9]. In addition, inductive elements were associated with the coexistence of mechanical movement (lateral squeezing, longitudinal stretching and pulling) during electrical activity in nerves [24].

In this manuscript we show that the appearance of inductive behavior in the nerve membrane as well as in pure lipid membranes may arise from the voltage and time dependence of membrane capacitance and membrane conductance. We will consider two types of experiments, namely impedance spectroscopy and voltage jump experiments, commonly performed in the field of electrophysiology. In the following two sections, we show that the common approximation that conductance and capacitance of the lipid membrane are constant, is far from valid, especially, in the vicinity of the lipid melting transition, and its validity is even questionable beyond this region.

### 1.1. Nonlinear Capacitance

Given the small bimolecular thickness and the low dielectric constant (ε = 2 to 4) as compared to that of the surroundings (ε≃ 80 for water), lipid bilayers are usually considered as capacitors when dealing with the electrical properties of both synthetic and biological membranes. Like commercial planar capacitors, they are able to store charges (ions) on the surface of their leaflets when a transmembrane potential difference is present. This is usually the case for plasma membranes under physiological conditions, where resting potentials arise due to uneven distributions of ions between the inside and the outside of the cell. They are of the order of 100 mV for a cell at rest. Unlike common commercial capacitors, however, where mechanical constraints counterbalance the attractive force between the oppositely charged plates, lipid membranes can significantly alter their dimensions (e.g., they are compressed) when an external voltage is applied. This effect is called electrostriction. As a result, the value of the capacitance changes as a nonlinear function of the applied voltage. This has been shown in theory [5] and in experiments, in which a quadratic dependence of the capacitance on the voltage was measured for both pure lipid bilayers [3] and human embryonic kidney cell (HEK) membranes [4].

The magnitude of the capacitance change depends on the elastic properties of the membrane and is expected to increase dramatically close to the melting transition, where the elastic constants are at maximum [5,25] and the membrane is softer. A lipid bilayer of DPPC increases its area by 24.6% and decreases its thickness by 16.3% in the transition from the gel to the fluid state [26]. For a planar capacitor ($C_{m}=\varepsilon A/d$) this corresponds to a capacitance of the fluid membrane which is ≃1.5 times larger than that of the gel phase (assuming a constant dielectric constant) [5]. The melting transition of lipid membranes is known to be affected by the change in several intensive thermodynamic variables like hydrostatic and lateral pressure, chemical potential, and, of special interest for the present treatment, voltage [7].

In addition, membranes can display a spontaneous polarization in the absence of an electric field if they contain dipole moments that are not counterbalanced through a geometrical, chemical or physical symmetry. This is usually the case for biological and lipid membranes, and the result is that the membrane can be charged in absence of an electric field (see [6] for details). The general expression for the charge on the membrane capacitor is given by,

(1) $$q_{C}=A\left(\varepsilon E+P_{0}\right)=\varepsilon \frac{A}{d}V+AP_{0}=C_{m}V+AP_{0}$$

where the capacitance ($C_{m}$), area (*A*), thickness (*d*) and offset polarization ($P_{0}$) of the membrane can all depend on the applied voltage (V=E·d), as well as on the state of the membrane, effectively making the charge a nonlinear function of the voltage (see Appendix for the details of the calculation). The last term represents the “offset charges” and can be different from zero in the absence of a voltage difference if the membrane displays a spontaneous polarization.

### 1.2. Nonlinear Conduction

The equivalent circuit representation of a membrane of Figure 1A is able to describe two fundamental functions of membranes: the storage of energy (represented by the capacitance $C_{m}$) and the conduction of ions (pictured by the resistance $R_{m}$ or, equivalently, by the conductance $G_{m}=1/R_{m}$). Traditionally, in the case of biological membranes the latter role is entrusted uniquely to protein channels, hence the dependence of the resistance on variables like time and voltage is a model for the specific opening and closing mechanism of such proteins. The same circuit (Figure 1A) is used to describe pure lipid membranes, with the main difference that the resistance represents the very small leak conduction through the bilayer, thus it has a constant and very small value (up to more than 3 orders of magnitude smaller than that of cell membranes [27]).

This scenario, however, is far from complete. Lipid membranes have been shown to be able to conduct ions in a fashion similar to the conduction of protein ion channels. The similarity includes several aspects of protein conduction: quantized conduction events, gating due to drugs, temperature, pH effects, ions (especially calcium) and voltage [8,28,29]. The lipid ion channels can be seen as defects in the membrane (pores) through which ions can pass [30].

The conduction of pure lipid membranes has been shown to follow the magnitude of the fluctuations of the membrane. Hence, conduction follows the excess heat capacitance profile of the membrane [31,32] and is maximum at the melting transition. The direct connection between the lipid melting transition and permeability of the membrane has been confirmed by Andersen *et al.* [33]. They showed that the membrane becomes highly permeable in the regions which are in the transition and has very low permeability outside this region (both for gel and fluid regions).

The simplest way of modeling the voltage dependence of the creation of a pore in the membrane is through electrostriction. The charges on the membrane capacitor squeeze the membrane until a pore is created and the tension can be relieved, which can be seen as local dielectric breakdown of the membrane. Since the membrane is softer in the transition, the creation of a pore will in general depend on voltage and on the state of the membrane. Using this simple model, Blicher and Heimburg very successfully described the current-voltage relationship measured in patch experiments on lipid membranes [29]. The current-voltage relationship of pure lipid membranes described in [29] very much resembles that of two types of TRP channels (Transient Receptor Potential Channel), showing both nonlinearity and rectification. Interestingly, data measured for TRP channels are likewise very well described by this simple capacitor model, suggesting a possible common mechanism at the base of ion conduction for lipid as well as biological membranes.

Lacking a detailed functional expression for the voltage and state dependence of the membrane conductance, we will here assume that it is well represented by the nonlinearity of the current-voltage relationships like the one measured in [29]. Of particular interest for the analysis of the electrical behavior of membranes, as we’ll see in the theory part, is the time dependence of the nonlinear behavior. Since the membrane conduction follows the magnitude of the membrane fluctuations, we can argue that the dynamics of the conduction must follow the relaxation dynamics of the membrane.

In this publication, we predict the electrical behavior of lipid membranes which display nonlinear capacitance and nonlinear conduction even in the absence of proteins. We do that for commonly performed experiments in electrophysiology and show that strong similarities exist between the response of biological and artificial membranes, especially close to their melting transition, suggesting that one should take this into account when interpreting the results of such experiments.

## 2. Theory

The electrical properties of biological membranes are commonly investigated through perturbation experiments, which can be grouped into two main classes: jump and sinusoidal perturbation techniques. We will here explore the implications of the nonlinear electrical properties of biological and artificial lipid membranes for the correct interpretation of the results of these two types of experiments. Specifically, we will consider the current response of a membrane due to a change in the applied voltage, the so-called voltage clamp [34]. In the following, the membrane is modeled as a resistor in parallel to a capacitor, as shown in Figure 1A.

Perturbation techniques do not only reveal the amplitude of the response of the investigated system, but also the dynamics of this response. The response of a system to a perturbation is described by response theory. For simplicity we limit our considerations to linear response theory, where the response depends linearly on the perturbation. The linear response of a system to a change in the applied voltage is given by,

(2) $$\alpha \left(t\right)=\int_{-\infty }^{t}\Gamma \left(t-t^{\prime }\right)\dot{V}\text{d}t^{\prime }$$

where α is the response, $\dot{V}=\text{d}V/\text{d}t^{\prime }$ is the rate of change in voltage and Γ=(∂α/∂V)(t) is the linear transfer function, connecting the change in voltage to the response. If the system under investigation is a capacitor, then the transfer function is related to the capacitive susceptibility, $\hat{C}=\partial q/\partial V$, and the response is the change in charges on the capacitor,

(3) $$\Delta q_{C}\left(t\right)=\int_{-\infty }^{t}\hat{C}\left(t-t^{\prime }\right)\dot{V}\text{d}t^{\prime }\;$$

While the capacitive susceptibility usually is an equilibrium property [5], it is here a function of time, *i.e.*, it may change after a perturbation due to time-dependent equilibration of the membrane porperties. The capacitive current is given by $I_{C}\left(t\right)=\text{d}\Delta q_{C}\left(t\right)/\text{d}t$. Considering only a resistor instead, the transfer function is the conductance, *G*, and the response is the resistive current,

(4) $$\Delta I_{\Omega }\left(t\right)=\int_{-\infty }^{t}G\left(t-t^{\prime }\right)\dot{V}\text{d}t^{\prime }$$

Note that the transfer functions depend on the time difference, such that the response functions (Equations (2)–(4)) assume the form of a convolution. We see that the response functions generate the formalism expected for classic linear electronics by assuming that the conductance and the capacitance are time independent (*i.e.*, $\hat{C}=C_{m}$):

(5) $$\begin{array}{ccc} \Delta I_{C}\left(t\right) & = & C_{m}\frac{\text{d}V(t)}{\text{d}t} \end{array}$$

(6) $$\begin{array}{ccc} \Delta I_{\Omega }\left(t\right) & = & G\Delta V(t) \end{array}$$

However, for biological as well as artificial lipid membranes, the capacitance and the conductance are known to be both voltage and time dependent. In the following, we consider the implications of these two dependencies.

The voltage dependence of the transfer functions implies that the response is not linear with respect to the perturbation, which is the working hypothesis of linear response theory. To overcome this, we limit ourselves to small voltage perturbations, for which the response can be assumed to be linear. The equilibrium change in the Ohmic current after a small change in voltage, $\Delta V=V-V_{h}$, from the holding voltage $V_{h}$ to voltage *V* is:

(7) $$\begin{array}{ccc} \Delta I_{\Omega } & ≃ & {\left(\frac{\partial I_{\Omega }}{\partial V}\right)}_{V_{h}}\Delta V={\left(\frac{\partial (G_{m}V)}{\partial V}\right)}_{V_{h}}\Delta V=\left(G_{m}\left(V_{h}\right)+{\left(\frac{\partial G_{m}}{\partial V}\right)}_{V_{h}}V_{h}\right)\Delta V\\ & \equiv & \underset{G}{\underset{︸}{\left(G_{0}+\Delta G_{0}\right)}}\Delta V \end{array}$$

Similarly, the equilibrium change in charge on the membrane capacitor after a small change in voltage from the voltage $V_{h}$ is given by

(8) $$\begin{array}{cc} \Delta q_{C} & ≃{\left(\frac{\partial q_{C}}{\partial V}\right)}_{V_{h}}\Delta V={\left(\frac{\partial (C_{m}V+AP_{0})}{\partial V}\right)}_{V_{h}}\Delta V=\\ & \left(C_{m}\left(V_{h}\right)+{\left(\frac{\partial C_{m}}{\partial V}\right)}_{V_{h}}V_{h}+{\left(\frac{\partial (AP_{0})}{\partial V}\right)}_{V_{h}}\right)\Delta V\equiv \underset{\hat{C}}{\underset{︸}{\left(C_{0}+\Delta C_{0}\right)}}\Delta V \end{array}$$

where $C_{0}$ is the constant value of the capacitance at the holding voltage and $\Delta C_{0}$ is due to changes in capacitance and polarization with voltage, and $\hat{C}=C_{0}+\Delta C_{0}$ is the capacitive susceptibility defined in [5,6].

We will now assume that the functions $\hat{C}$ and *G* defined above are time dependent because after a voltage jump membranes need to relax into a new state with a characteristic time constant. We will approximate this time dependence with a single exponential relaxation, such that transfer functions take the forms:

$$\begin{array}{ccc} \hat{C}\left(t-t^{\prime }\right) & \approx & C_{0}+\Delta C_{0}\left(1-e^{-\frac{t-t^{\prime }}{\tau }}\right) \end{array}$$

(9) $$\begin{array}{ccc} G(t-t^{\prime }) & \approx & G_{0}+\Delta G_{0}\left(1-e^{-\frac{t-t^{\prime }}{\tau }}\right) \end{array}$$

Both $C_{0}$ and $\Delta C_{0}$ (as well as $G_{0}$ and $\Delta G_{0}$) depend on the holding voltage, $V_{h}$. Changes in the capacitive susceptibility are coupled to mechanical and thermodynamical changes, and these, in turn, can also affect both the capacitance and the conductance [6].

Using Equation (9) the capacitive and ohmic current for small changes in voltage assume the following form:

(10) $$\Delta I_{C}\left(t\right)=C_{0}\frac{\text{d}V(t)}{\text{d}t}+\Delta C_{0}\frac{\text{d}}{\text{d}t}\left(\int_{-\infty }^{t}\left(1-e^{-\frac{t-t^{\prime }}{\tau }}\right)\dot{V}\left(t^{\prime }\right)\text{d}t^{\prime }\right)$$

(11) $$\Delta I_{\Omega }\left(t\right)=G_{0}\Delta V\left(t\right)+\Delta G_{0}\int_{-\infty }^{t}\left(1-e^{-\frac{t-t^{\prime }}{\tau }}\right)\dot{V}\left(t^{\prime }\right)\text{d}t^{\prime }$$

### 2.1. Impedance Spectroscopy

In the following we consider two types of electrical perturbation techniques. The first type consists of a sinusoidal perturbation. In impedance spectroscopy, the membrane is perturbed by a small amplitude sinusoidal voltage and the amplitude and the phase of the response current is measured in order to determine the impedance, *Z*.

(12) $$Z\left(\omega \right)\equiv \frac{V(\omega )}{I(\omega )}=R\left(\omega \right)+iX\left(\omega \right)\;$$

where the real part (*R*) is the resistance and the imaginary part (*X*) is the reactance (Note that the real part of the impedance is conventionally called resistance, but it doesn’t necessary correspond to a physical resistor in the circuit of the system under investigation. The two coincide only for specific circuit configurations, e.g. a series combination of a resistor and a capacitor). By only applying low amplitude perturbations, impedance spectroscopy effectively linearizes the electrical response of the investigated system and we can apply linear response theory Equations (10) and (11). By Fourier transforming (Equations (10) and (11)) and using that the applied voltage is sinusoidal, we find the frequency dependent capacitive and resistive current,

(13) $$\Delta I_{C}\left(\omega \right)=\left(i\omega C_{0}+\Delta C_{0}\frac{i\omega }{1+i\omega \tau }\right)V\left(\omega \right)$$

(14) $$\Delta I_{\Omega }\left(\omega \right)=\left(G_{0}+\Delta G_{0}\frac{1}{1+i\omega \tau }\right)V\left(\omega \right)$$

where V(ω) is the Fourier transform of the applied voltage. From this we can calculate the impedance in accordance with Equation (12),

(15) $$Z_{C}\left(\omega \right)={\left(i\omega C_{0}+\Delta C_{0}\frac{i\omega }{1+i\omega \tau }\right)}^{-1}$$

(16) $$Z_{\Omega }\left(\omega \right)={\left(G_{0}+\Delta G_{0}\frac{1}{1+i\omega \tau }\right)}^{-1}$$

We see again that for constant capacitance and conductance the classical impedance of a capacitor and a resistor is recovered. The equivalent circuit for a membrane is a resistor in parallel with a capacitor (Figure 2A). Taking into account that the total impedance of parallel components is inverse of the sum of the inverses of the components impedances, the impedance of the membrane takes the form,

(17) $$\begin{array}{ccc} Z(\omega ) & = & {\left(\frac{1}{Z_{C}\left(\omega \right)}+\frac{1}{Z_{\Omega }\left(\omega \right)}\right)}^{-1} \end{array}$$

(18) $$\begin{array}{ccc} & = & {\left(i\omega C_{0}+\Delta C_{0}\frac{i\omega }{1+i\omega \tau }+G_{0}+\Delta G_{0}\frac{1}{1+i\omega \tau }\right)}^{-1} \end{array}$$

**Figure 3:**
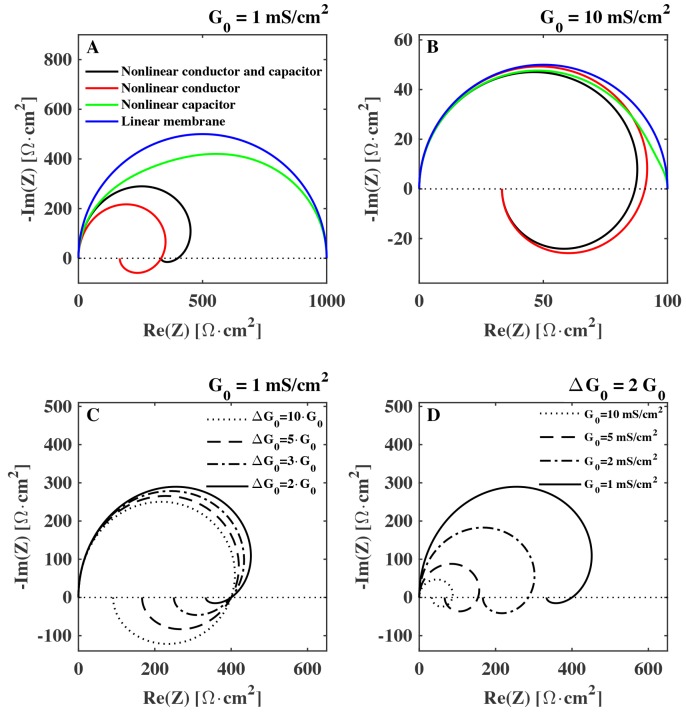
Calculated Nyquist plot of the impedance of a biological membrane (Equation (17)) shown for different degrees of nonlinearity: (**A**)–(**B**): **blue**: $\Delta G_{0}=0$ and $\Delta C_{0}=0$, **green**, $\Delta G_{0}=0$ and $\Delta C_{0}=0.5\cdot C_{0}$, **red**, $\Delta G_{0}=2\cdot G_{0}$ and $\Delta C_{0}=0$, **black**, $\Delta G_{0}=2\cdot G_{0}$ and $\Delta C_{0}=0.5\cdot C_{0}$. Membrane background conductance: $G_{0}=1\;mS/cm^{2}$ [1,9] (**A**), $G_{0}=10\;mS/cm^{2}$ (**B**). Membrane capacitance is $C_{0}=1\;\mu F/cm^{2}$ and the characteristic relaxation time is τ=1 ms. (**C**): different values of $\Delta G_{0}$ . (**D**): different values of membrane conductance $G_{0}$.

In Figure 3 the impedance of a membrane with capacitance $C_{0}=1$ µF/cm${}^{2}$ and relaxation time τ=1 ms is shown for different sets of biologically relevant values for $G_{0}$ and $\Delta G_{0}$ in a Nyquist plot. We see that especially the nonlinearity of the conductance can have a strong impact on the impedance. It is responsible for the spiraling in the Nyquist plot commonly associated to inductance. Specifically, we see that while the value of the background conductance $G_{0}$ has an influence on the whole impedance spectrum (Figure 3D), it is the value of the conductance change $\Delta G_{0}$ that sets the magnitude of the negative reactance (Figure 3C). On the other hand, the non-linearity of the capacitance has a more subtle effect on the complex component of the impedance. The effect of the nonlinearity of the capacitance is described further in the Discussion section.

### 2.2. Voltage Jumps

The second type of commonly performed electrical perturbation experiments is jump experiments. Using voltage-jump experiments, Hodgkin and Huxley characterized the electrical response of the squid giant axon, enabling them to propose their model for the action potential [1,2]. The voltage-jump technique is widely used to characterize the functionality of protein ion channels. Understanding the response of the membrane is therefore essential for correctly associating function to specific proteins.

**Figure 4:**
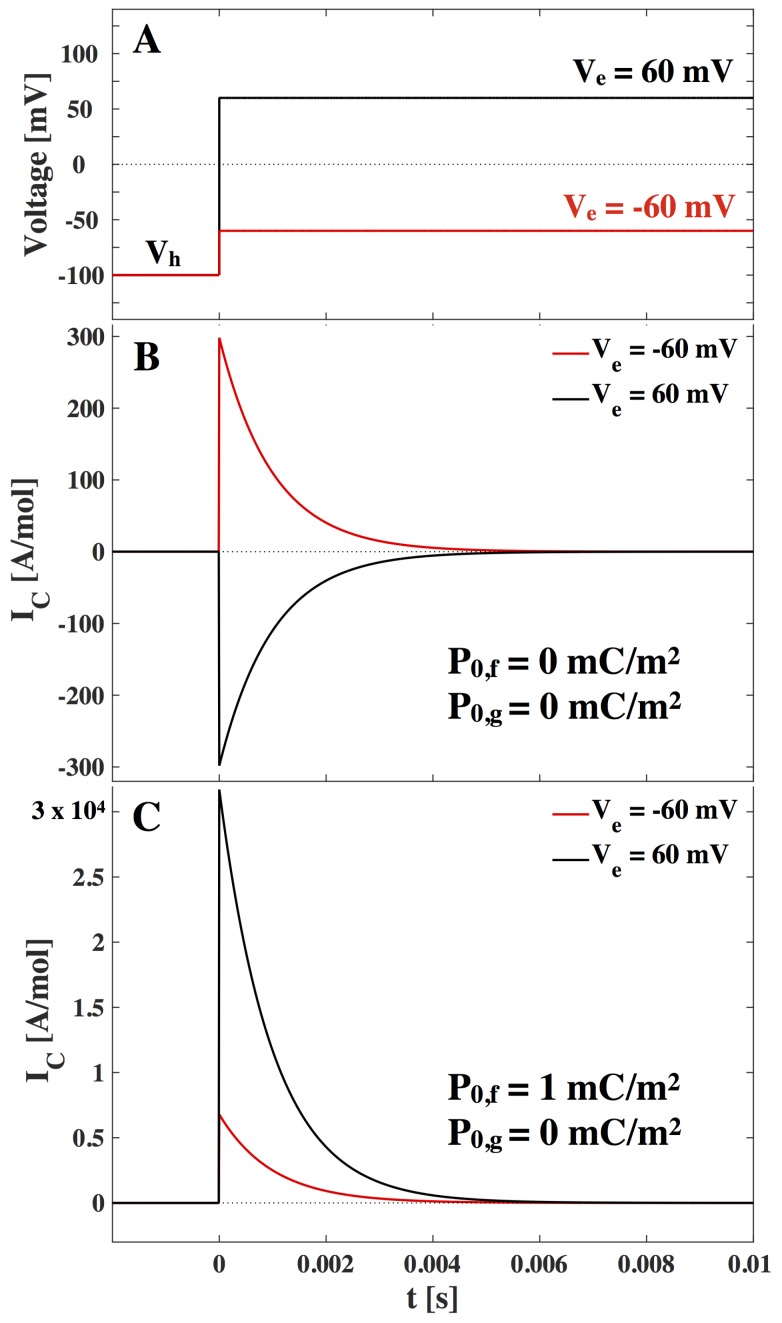
(**A**). Voltage jump at time *t* = 0 from a holding voltage of $V_{h}=-100\;$mV to an end voltage of $V_{e}=-60\;$mV (ΔV=40mV, **red**) and $V_{e}=+60\;$ mV (ΔV=160mV, **black**). (**B**)–(**C**). The capacitive current response to the voltage jump. (**B**). shown for membrane with no offset polarization assumed. **(C)**. shown for a polar membrane with spontaneous polarization $P_{0,f}=1\;$mC/m${}^{2}$ in the fluid phase and $P_{0,g}=0\;$mC/m${}^{2}$ in the gel phase. Values used are from LUV of DPPC (see Appendix), the temperature is T=314.5K and τ=1ms is assumed.

A voltage jump experiment is carried out by performing a fast jump in voltage, from a holding voltage ($V_{h}$) to end voltage ($V_{e}$). The current response of the system (the membrane) is recorded. Note, that we include in our consideration that the system can be kept at a holding voltage different from zero. We assume the jump to be performed at t=0 and that any change in the applied voltage is instantaneous. In dealing with impedance spectroscopy we could apply linear response theory since only small perturbations were considered and we could linearize the response. In the case of jump experiments we are not limited to small perturbations and the response of the membrane is not guaranteed to be linear. We, however, assume that equilibration of the membrane, its capacitance and polarization, follow single exponential relaxation. Using these assumptions we can calculate the time dependent change in the charges on the membrane capacitor due to a voltage jump $\Delta V=V_{e}-V_{h}$,

(19) $$\Delta q_{C}\left(t\right)=C_{m}\cdot \Delta V+\left(\Delta C_{m}V_{e}+\Delta \left(AP_{0}\right)\right)\left(1-e^{-\frac{t}{\tau }}\right)\;$$

where $C_{m}=C_{m}\left(V_{h}\right)$ is the capacitance before the voltage jump. $\Delta (AP_{0})$ is the voltage dependent change in offset charges (*i.e.*, a movement of charges fixed on membrane molecules). Instantaneous voltage changes cannot be practically applied in experiments. We have here used this idealization to simplify the response dynamics and we justify it with the observation that in experiments the capacitive current spike due to the constant capacitance is often much faster than other timescales under considerations and is often removed with compensation circuitry. Therefore we remove the initial capacity spike by only considering t>0. We calculated the changes in capacitance and polarization due to voltage changes in [6]. The capacitive current is then given in Figure 4:

(20) $$I_{C}\left(t\right)=(\left(\Delta C_{m}V_{e}+\Delta \left(AP_{0}\right)\right)\frac{e^{-\frac{t}{\tau }}}{\tau }$$

The current in Figure 4 is given in the units [A/mol] (mole of lipid, 1 A/mol ∼2/3 nA/cm${}^{2}$, assuming an area per mole of lipid of $A∼1.5\cdot 10^{5}$ m${}^{2}$/mol [25]). From this and Figure 4 we can expect current responses of up to 20 µA/cm${}^{2}$ after a voltage jump, which originate from the nonlinearity of the lipid membrane. Note that these values highly depend on the vicinity of the lipid melting transition. At the center of the transition we can expect responses up to 60 µA/cm${}^{2}$. We further expect that the current amplitude is inversely proportional to the characteristic relaxation time. We realize that the capacitive non-linear current response after a voltage jump can be significant when compared to response of electrophysiological recordings. In particular, they are of similar order to gating currents, which will be considered in the Discussion.

We now consider the dynamics of the conduction through the lipid membrane as a relaxation between two equilibrium states. We also assume that the equilibration follows the equilibration dynamics of the lipid membrane. With these assumptions, the resistive current response to a voltage jump assumes the following form:

(21) $$I_{\Omega }\left(t\right)=\left(G_{m}+\Delta G_{m}\left(1-e^{-\frac{t}{\tau }}\right)\right)V_{e}\;$$

where $G_{m}=G_{m}\left(V_{h}\right)$ is the background conduction or background leak, and $\Delta G_{m}$ is the change in conduction as a response to the voltage jump.

Using Equations (20) and (21), we can write the current response of the lipid membrane in the vicinity of the lipid melting transition to a voltage jump (for t>0) as,

(22) $$\begin{array}{cc} I_{m}\left(t\right)= & \left(G_{m}+\Delta G_{m}\left(1-e^{-\frac{t}{\tau }}\right)\right)V_{e}\\ & +\left(\Delta C_{m}V_{e}+\Delta \left(AP_{0}\right)\right)\frac{\exp\left(-\frac{t}{\tau }\right)}{\tau }\; \end{array}$$

**Figure 5:**
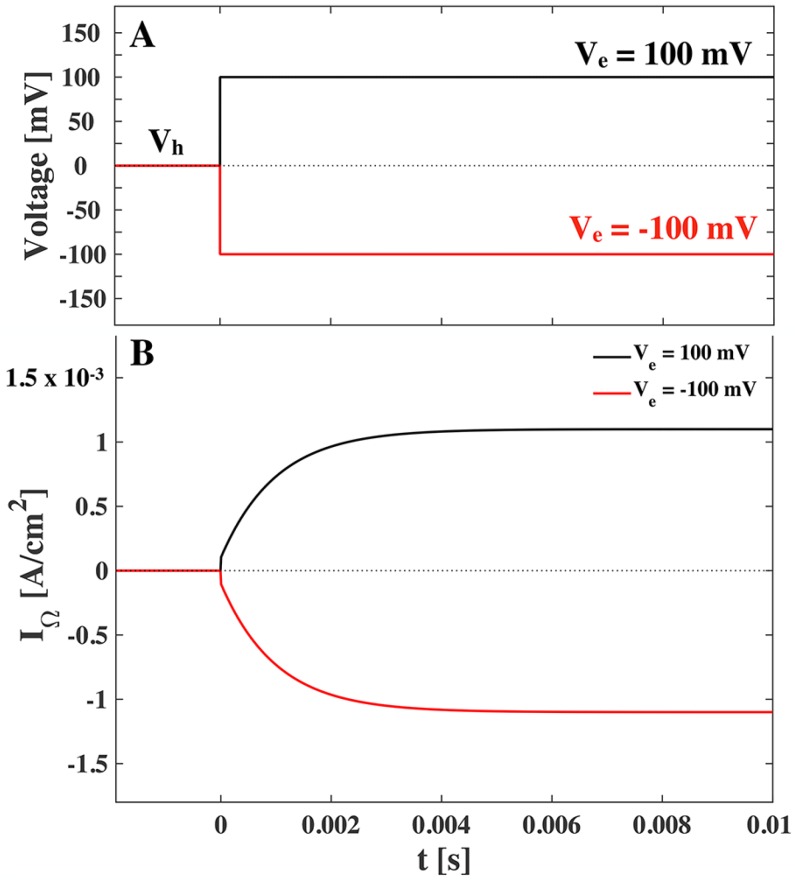
(**A**). Voltage jumps at *t*= 0 from a holding voltage of $V_{h}=0$ to an end voltage of $V_{e}=100\;$ mV (positive jump, black) and $V_{e}=-100\;$ mV (negative jump, red). (**B**). The resistive current response to positive and negative voltage jump according to Equation (21). The assumed conductance is $G_{0}=1\;mS/cm^{2}$ and $\Delta G_{m}=10\;$mS/cm${}^{2}$, and the characteristic relaxation time is τ=1 ms. No polarization or holding voltage is assumed.

Note the functional similarities between conduction and the nonlinear capacitance contribution. In the literature we find that biological membranes have a background conductance of around $G_{m}∼1\;$mS/cm${}^{2}$ and open channel conductance of around $\Delta G_{m}∼10\;$mS/cm${}^{2}$ [1,9]. The resistive current through the membrane (Equation (21)) is shown in Figure 5, where we have assumed a characteristic relaxation time τ=1ms.

When comparing Figure 4 and Figure 5, we recognize that the resistive current is around 500 times greater than the peak of the capacitive current. We note though that the capacitive current is inversely proportional to the relaxation time and hence if we assume a characteristic relaxation time of τ=0.1ms we see a ten-fold increase in the capacitive current.

## 3. Summary and Discussion

In their model for the propagation of nerve signals in giant squid axons, Hodgkin and Huxley [2] suggested that the ion channel proteins driving the signal must have a voltage-dependent gating mechanism. They proposed that this gating mechanism would originate from a charged part of the protein moving in the electric field, which would generate a small capacitive current signal (gating current). Applying various experimental tricks, mainly involving lowering the conductive currents of the nerve membrane, a small capacitive current after a voltage jump has in fact been found experimentally, showing a maximum amplitude of about 30 µA/cm${}^{2}$ and temporal width of around 0.1 ms [35,36]. In their model, Hodgkin and Huxley assumed that the capacitance of the membrane in nerves is constant. This is, however, not the case close to transitions [5]. Voltage applied across membranes can change the physical state of the lipid membrane. This leads to a non-linear response of the membrane capacitance on applied voltage. Here, we demonstrated that the nonlinear capacitance of the membrane itself can lead to a variety of current responses after a voltage jump, depending on the physical state of the membrane (gel, fluid or in the transition regime) and its spontaneous polarization [6]. Since the relaxation time of membranes is of the order of 1 ms to 100 ms, these nonlinear capacitive currents are temporally separable from the current due the constant part of the capacitance. Blatt [37] suggested already in 1977 that experimentally observed gating currents could be due to the nonlinear voltage-dependence of the membrane capacitor rather than to the movement of gating charges. By only considering the electrostrictive effect, Blatt found that the nonlinear capacitive current was of the same order of magnitude as the gating current, though in the opposite direction (as already pointed out by Keynes and Rojas [36]). Here we calculated the same direction for the nonlinear capacitive current as Blatt, if we only consider electrostriction. However, in the presence of a permanent polarization of the membrane, the gating current and the nonlinear capacitive current may have the same direction. Thus, excluding the possibility that the recorded gating current is due to nonlinear capacitive currents of the membrane is no longer possible. A more detailed investigation is needed. The biological relevance of our calculations is underlined by the experiments by Farrell *et al.* [4], which show significant non-linearity of the capacitance of HEK cells (human embryonic kidney) including offset polarization effects following our predictions.

It was shown by [7,8,29,31] that lipid membranes display also a high conductivity close to the lipid melting transition. Since the membrane state responds to changes in voltage, the conductance also displays non-linear behavior. Furthermore, the membrane ion channels display similar conduction features as those commonly associated to specific ion channel proteins. This implies that lipid membranes close to transition display channel-opening and closing events similar to protein channels. The similarities include voltage-gating, response to drugs (anesthetics [31]) and temperature-dependent conductivity. Any factor which influences the lipid melting transition will influence the membrane conduction [32]. Arguing that the membrane conduction must follow the dynamics of the fluctuations of the lipid membrane (due to the fluctuation-dissipation theorem, fluctuations are large and slow in the transition regime) we showed that the temporal conduction behavior (open lifetimes) of lipid ion channels is similar to the behavior of voltage-gated proteins (such as the potassium channel first investigated by Hodgkin and Huxley [1,38]). Lipid ion channels do not only display conduction amplitudes similar to those of proteins. Their dynamical properties mimic the protein ion channel dynamics, both on macroscopic level (discussed here) and on a microscopic level [29]. The similarity between the conduction of lipid channels and protein channels fundamentally complicates the association of conduction properties with a specific protein. Here, we associated the conductance of a membrane to the conductivity of the lipid membrane itself. The time-scale of channel-opening is related to the fluctuation lifetimes as studied by [39,40]. Thus, many prominent features of the biological membrane can in fact originate from the lipid bilayer itself.

We demonstrated that the electrical impedance of lipid membranes can show a negative reactance at low frequencies when the time dependence of the nonlinearity of the conductance is considered. The negative reactance (see Figure 3) leads to a spiraling shape that qualitatively resembles the Nyquist plots measured previously for the squid giant axon (Figure 2B) [17]. The spirals in the Nyquist plots were tentatively explained by Cole and Curtis (1939) by assuming the presence of an inductive element in the circuit representation of the membrane (Figure 1B). By assuming a single exponential relaxation dynamics for both membrane capacitance and conductance, we could reproduce the inductive behavior by modeling the membrane by a circuit element with parallel resistor and capacitor, without the necessity of adding an actual inductor into the equivalent circuit of the membrane. An inductance in the membrane of approximately 0.2 H cm${}^{2}$ as the one estimated in [11] is difficult to explain since it is not suggested by any structural property of the membrane. In particular, there exist no conducting coils in the membrane. The suggestion of a membrane inductance was exclusively made based on the analysis of electrical measurements.

Impedance spectroscopy is a very powerful tool to characterize material properties, but its data require detailed information about the structure of the system under investigation in order to be correctly interpreted. The reason for this is that there is no unique electric circuit, which can explain a particular impedance spectrum. The choice of a specific circuit element or circuit configuration must therefore be justified by structural information. For this reason, impedance spectroscopy is often accompanied by other complementary techniques (e.g., SEM, TEM) and this explains the reluctance in introducing an inductive element in the equivalent circuit of biological membranes.

Following this line of reasoning, Cole [9] noted that inductive behavior is not necessarily nor uniquely explained by the ability of a system to store magnetic energy. Any system for which the potential difference is proportional to the rate of change of current (ΔV∝dI/dt) does, in fact, have the electrical properties of an inductance, and this is the only direct information that can be extracted by the impedance spectrum of Figure 2B. Any circuit in which dI/dt=f(V) will contain an element that can be (mis)-interpreted as an inductance. This can be any kind of process where the conductance of a system changes with time. Interestingly, among the various physical processes with this property, Cole mentioned processes which involve couplings of electrical properties and thermal or mechanical properties (thermoelectricity and piezoelectricity) [9]. He discarded them because he expected that such changes would be very small. However, the structure of membranes, and the presence of phase transition in biological membranes was not known at that time. Lipid membranes are now known to display interesting electromechanical properties (e.g., piezoelectricity, flexoelectricity), whose magnitude is greatly enhanced in the melting transition. This must be taken into account when interpreting electrical measurements. Here we showed how it is mostly the voltage induced change of conductance that affects the “inductive” part of the impedance spectrum (see Figure 3C). We therefore expect the effect to be maximum close to the melting transition where the permeability is enhanced and the membrane is more susceptible to small changes in voltage. Since biological membranes typically display a melting transition a few degrees below the physiological temperature (e.g., [41,42]), the nonlinearity of the membrane capacitance and conductance must be included in the interpretation of electrical measurements of biological membranes.

## 4. Conclusions

We have shown that in the vicinity of the lipid melting transition the commonly made assumption that the electrical properties of the lipid membrane are constant is an oversimplification. The capacitance of the membrane is nonlinear. The spontaneous polarization of asymmetric membranes introduces an extra level of nonlinearity, both of which are time dependent after a perturbation. Further, response of the conductivity of the lipid membrane to a perturbation is also nonlinear, and the nonlinearity is time dependent. Such phenomena can be wrongly associated to an inductive element in the membrane, leading to a negative reactance of the membrane at low frequencies. Such a negative reactance was described for the squid axon. Interestingly, the capacitive currents of the membrane after a perturbation are strikingly similar to gating currents**;** the conduction through the membrane shows great similarities with the conducting properties commonly associated to protein ion channels.

## Appendix

The explicit temperature and voltage dependence of the membrane capacitance and polarization has been calculated following [6]. There, the cooperative melting transition of the membrane from the gel to the fluid state is modeled as a two-state transition governed by a van’t Hoff law. In particular, the free energy difference between the two states in the presence of an applied voltage is given by,

(23) $$\Delta G\left(T,V\right)=\left(\Delta H_{0}-T\Delta S_{0}\right)-\frac{\bar{\Delta C_{m}}}{2}V^{2}-\bar{\Delta (AP_{0})}V$$

where $\bar{\Delta C_{m}}$ and $\bar{\Delta (AP_{0})}$ are the differences in the capacitance and the offset charge between the fluid and the gel state when V=0. For LUV of DPPC the melting enthalpy and entropy was $\Delta H_{0}=39$ kJ·mol${}^{-1}$ [5] and $\Delta S_{0}=124.14$ J·mol${}^{-1}$· K${}^{-1}$ (given a melting temperature $T_{m}=314.15$ K [6]). The thickness in the gel and in the fluid state are $d_{g}=4.79$ nm and $d_{f}=3.92$ nm, respectively [25]. The area per lipid in the gel state is $A_{g}=0.474$ nm${}^{2}$ and in the fluid state is $A_{f}=0.629$ nm${}^{2}$ [25]. Thus the difference in the capacitance between the two states (in the absence of voltage) is $\bar{\Delta C_{m}}=655.85$ F·mol${}^{-1}$, assuming a dielectric constant of $\varepsilon =4\varepsilon_{0}$ ($\varepsilon_{0}$ is the vacuum permittivity). The spontaneous polarization $P_{0}$ was assumed to be either zero, or (for Figure 4C), values for the fluid and the gel state are $P_{0g}=0$ mC·m${}^{-2}$ and $P_{0f}=1$ mC ·m${}^{-2}$.

Assuming a cooperative unit size of n=170 the equilibrium constant between the gel and fluid state and the average fraction of the lipids in the fluid state, $f_{f}\left(T,V\right)$, can be calculated (see Equations (37)–(39) in [6]). The area of the membrane is described by $A\left(T,V\right)=A_{g}+f_{f}\Delta A$, the thickness by $d\left(T,V\right)=d_{g}-f_{f}\Delta d$, and the spontaneous polarization by $P_{0}\left(T,V\right)=P_{0g}+f_{f}\Delta P_{0}$. The voltage and temperature dependence of the membrane capacitance is then given by $C_{m}\left(T,V\right)=\varepsilon A\left(T,V\right)/d\left(T,V\right)$.
